# Hospitals and Hospital Visitors

**Published:** 1909-10-30

**Authors:** 


### HOSPITALS AND HOSPITAL VISITORS.

*To the Editor of* The Hospital.

"Superintendent" writes: I can match Mr. Sydney Holland's experience of the undesirable results sometimes accruing from too much liberty being allowed as regards visitors to patients. Some years ago, in a male adult ward at a London hospital, a young married lady of position was in the habit of coming almost weekly to see the patients. She would read to them, bring them flowers, and so forth. It was early noticed that while the "daddies" were severely neglected, a young and good-looking man was sure of specially sympathetic attention. Not much was thought of this until we found that the gossip of the ward concerned itself largely with this lady, and seemed in possession of many details concerning her married life; how, for instance, that she was married to a man double her age, for whom she felt very little affection. Just a little later I ascertained that a former patient, a young non-commissioned officer in the Army, had accepted an invitation to lunch with the lady in question at a restaurant. The higher hospital powers were then invoked, and the visits suddenly ceased. The moral of this is surely that co-operation among all office-bearers in a hospital is necessary in the matter under notice, as, indeed, is everything touching their institution.

